# Effects of eliminating interactions in multi-layer culture on survival, food utilization and growth of small sea urchins *Strongylocentrotus intermedius* at high temperatures

**DOI:** 10.1038/s41598-021-94546-1

**Published:** 2021-07-23

**Authors:** Fangyuan Hu, Xiaomei Chi, Mingfang Yang, Peng Ding, Donghong Yin, Jingyun Ding, Xiyuan Huang, Jia Luo, Yaqing Chang, Chong Zhao

**Affiliations:** Key Laboratory of Mariculture and Stock Enhancement in North China’s Sea, Ministry of Agriculture and Rural Affairs, Dalian Ocean University, Dalian, 116023 China

**Keywords:** Marine biology, Fisheries

## Abstract

Poor growth and disease transmission of small sea urchins *Strongylocentrotus intermedius* in summer greatly hamper the production efficiency of the longline culture. Reducing the adverse effects of high stocking density while maintaining high biomass is essential to address these problems. Here, we conducted a laboratory experiment to simulate the multi-layer culture for sea urchins at ambient high temperatures (from 22.2 to 24.5 °C) in summer for ~ 7 weeks. Survival, body size, lantern growth, gut weight, food consumption, Aristotle's lantern reflex, 5-hydroxytryptamine concentration, pepsin activity and gut morphology were subsequently evaluated. The present study found that multi-layer culture led to significantly larger body size than those without multi-layer culture (the control group). This was probably because of the greater feeding capacity (indicated by lantern growth and Aristotle's lantern reflex) and food digestion (indicated by morphology and pepsin activity of gut) in the multi-layer cultured sea urchins. These results indicate that multi-layer is an effective approach to improving the growth efficiency of sea urchins at high temperatures. We assessed whether eliminating interaction further improve these commercially important traits of sea urchins in multi-layer culture. This study found that eliminating interactions displayed greater body size and Aristotle's lantern reflex than those not separated in the multi-layer culture. This approach also significantly reduced the morbidity compared with the control group. These novel findings indicate that eliminating interactions in multi-layer culture greatly contributes to the growth and disease prevention of sea urchins at high temperatures. The present study establishes a new technique for the longline culture of sea urchins in summer and provides valuable information into the longline culture management of other commercially important species (e.g. scallops, abalones and oysters).

## Introduction

The sea urchin *Strongylocentrotus intermedius* is a commercially important species widely cultured in China^1^ and Japan^2,3^. The annual production of sea urchins was over 8242 tons in China in 2019^4^. Longline culture is the most important approach to developing *S*. *intermedius* aquaculture^5^. It takes ~ 2 years for *S*. *intermedius* to develop into the market size (> 5 cm test diameter) from fertilized eggs. Small sea urchins (~ 3 cm test diameter) experience one summer and are subsequently harvested before the following summer in longline culture^6^. However, increasing the supply of cultured sea urchins is seriously endangered by high temperatures. The slow growth of *S*. *intermedius* in summer, e.g. 2.5 mm per month in northern China^7^, leads to the inefficiency of sea urchin aquaculture^5,8^. In addition, disease transmission deteriorates the production in summer^9–12^. Little information is available on how to improve growth and prevent disease transmission of sea urchins in longline culture in summer, leaving the most serious problem of the sea urchin aquaculture unaddressed.

High stocking density links to the economic viability or profitability of intensive aquaculture practices^13,14^. Increased stocking density, however, has adverse effects on the survival and growth performance of sea urchins^15^. The general solution is to reduce the stocking density^16^, but this inevitably decreases the profitability of sea urchin aquaculture. Establishing a method, which can avoid or reduce the adverse effects of high stocking density while maintaining high biomass, is therefore important for sea urchin aquaculture. Interestingly, there are two definitions of density in aquatic animals due to their different range of activities. For example, kg/m^3^ is used for most fishes^17^, while kg/m^2^ is for the benthic animals (e.g. sea urchin)^18,19^. This suggests that multi-layer culture (i.e. reduced density of each layer with no change of biomass) for sea urchins is theoretically valuable, although environmental sustainability is not taken into account. It is worthwhile to investigate whether multi-layer culture improves the survival, food utilization and growth of *S*. *intermedius* in longline culture at high temperatures.

Further, interaction of individuals increases with increasing stocking density of sea urchins, resulting in the competition for food and space, aggression and even cannibalism^18,20,21^. High interaction not only heightens the risk of disease transmission within colonies^22^, but displays adverse effects on the growth of aquatic species, including *S*. *intermedius*^16^. Eliminating interactions among individuals at high stocking density did not negatively alter the gonad index of sea urchins^23^. We consistently found that interactions seriously hamper the movement of *S*. *intermedius*^24^. Thus, it is reasonable to hypothesize that eliminating interactions in multi-layer culture probably further improves important traits of sea urchins.

The main purpose of this study is to explore an effective approach to improving the survival and growth of small sea urchins of longline culture in summer. We ask (1) whether multi-layer culture improves the survival, food utilization and growth of sea urchins; (2) whether eliminating interaction in multi-layer culture contributes to further improvement of these important traits of sea urchins.

## Results

### Mortality, morbidity and rearing space

**Figure 1:**
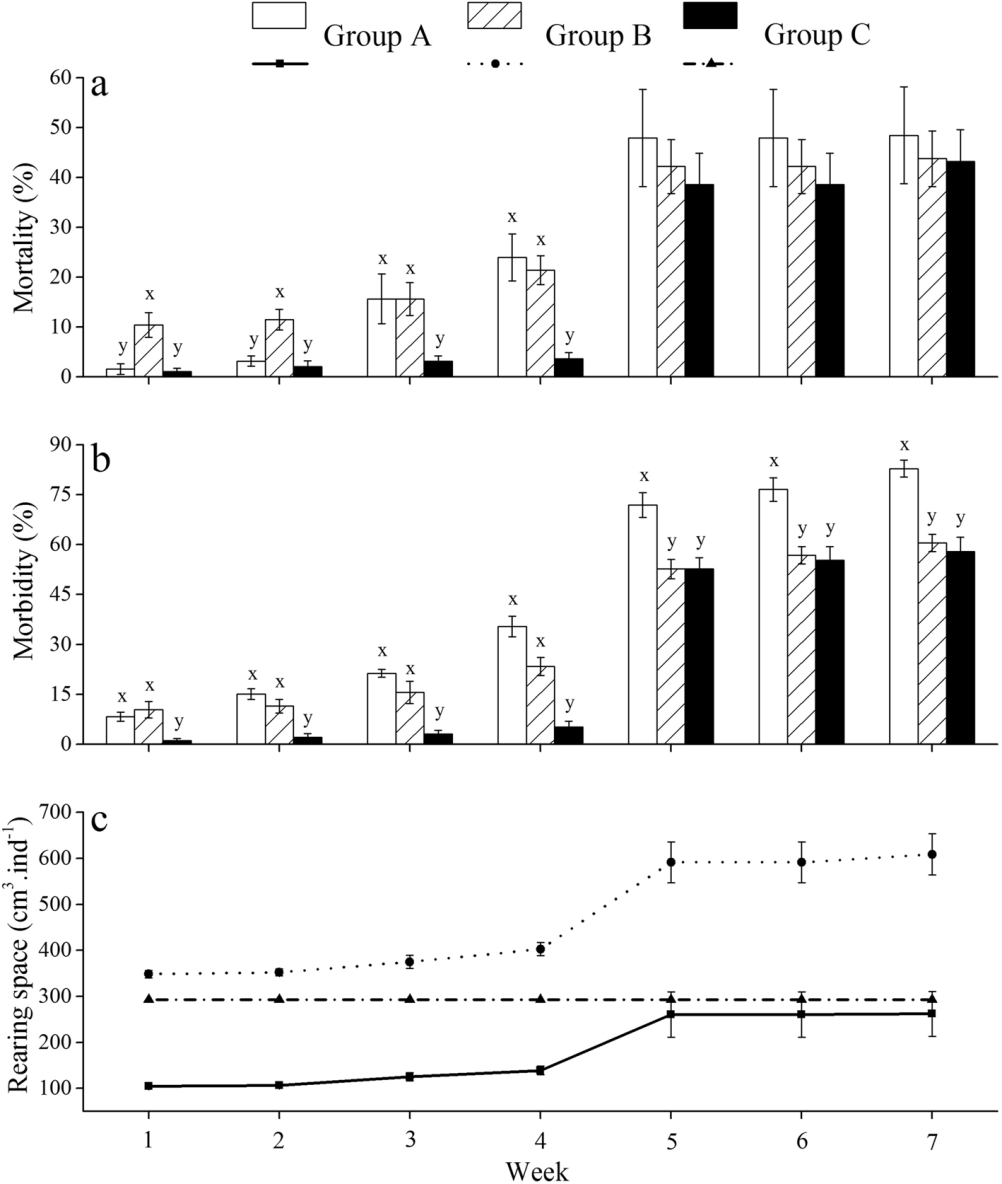
Mortality (**a**) and morbidity (**b**) and rearing space (**c**) of *Strongylocentrotus intermedius* of the experimental groups during the experiment (mean ± SD, N = 8). Letters above the bars represent significance in each week (*P* < 0.05).

There was no significant difference in mortality between groups A and B (all *P* > 0.05), except for those in week 1 and week 2 (both *P* < 0.05) (Fig. 1a; Supplementary Table 1).

The mortality in group C was significantly lower than that in group B in weeks 1, 2, 3, 4 (all *P* < 0.05), but showed no significant difference in weeks 5, 6, 7 (all *P* > 0.05) (Fig. 1a; Supplementary Table 1).

Morbidity in group A was significantly higher than that in group B in weeks 5, 6, 7 (all *P* < 0.05), but not significantly different in weeks 1, 2, 3, 4 (all *P* > 0.05) (Fig. 1b; Supplementary Table 1).

Significantly higher morbidity was detected in group B than that in group C in weeks 1, 2, 3, 4 (all *P* < 0.05) but not in weeks 5, 6, 7 (all *P* > 0.05) (Fig. 1b; Supplementary Table 1).

With the increase in mortality, the rearing space of groups B and A increased (139.3 ± 26.0 cm^3^ ind^−1^ for group A and 402.9 ± 70.2 cm^3^ ind^−1^ for group B in week 4) (262.2 ± 137.5 cm^3^ ind^−1^ for group A and 608.8 ± 217.8 cm^3^ ind^−1^ for group B in week 7). The rearing space of group C was maintained at 297.4 cm^3^ ind^−1^ (Fig. 1c).

### Food consumption

**Figure 2:**
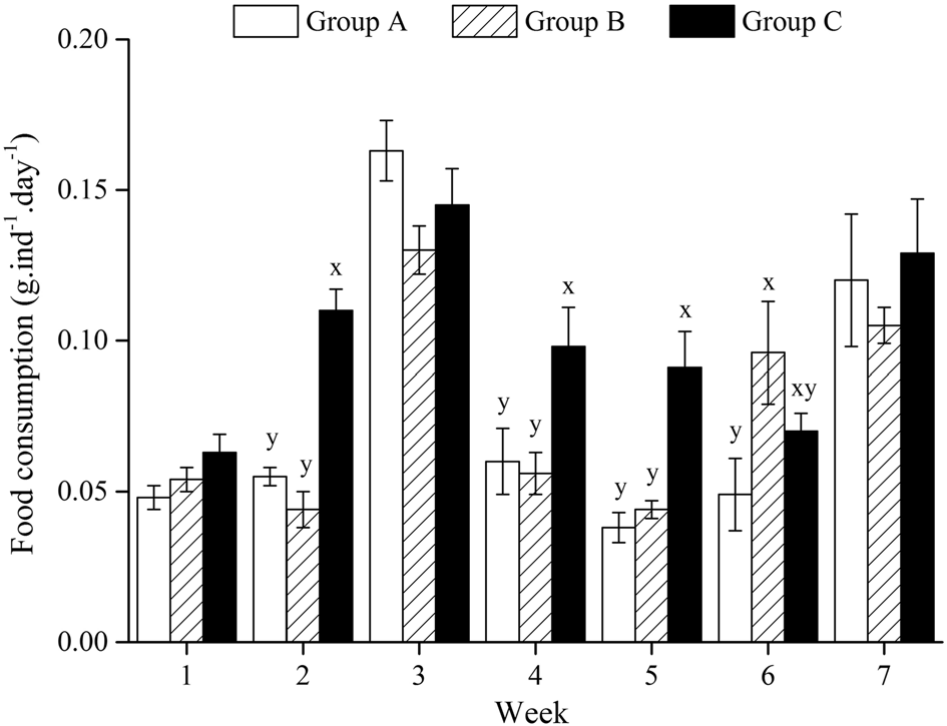
Food consumption (g dry weight) of *Strongylocentrotus intermedius* of the experimental groups in 7 weeks (mean ± SD, N = 8). Letters above the bars represent significance in each week (*P* < 0.05).

There was no significant difference in food consumption between groups A and B in this experiment (all *P* > 0.05), except for that in week 6 (*P* < 0.05). The food consumption in group C was significantly larger than that in group B in weeks 2, 4, 5 (all *P* < 0.05) (Fig. 2; Supplementary Table 1).

### Test diameter and body weight

**Figure 3:**
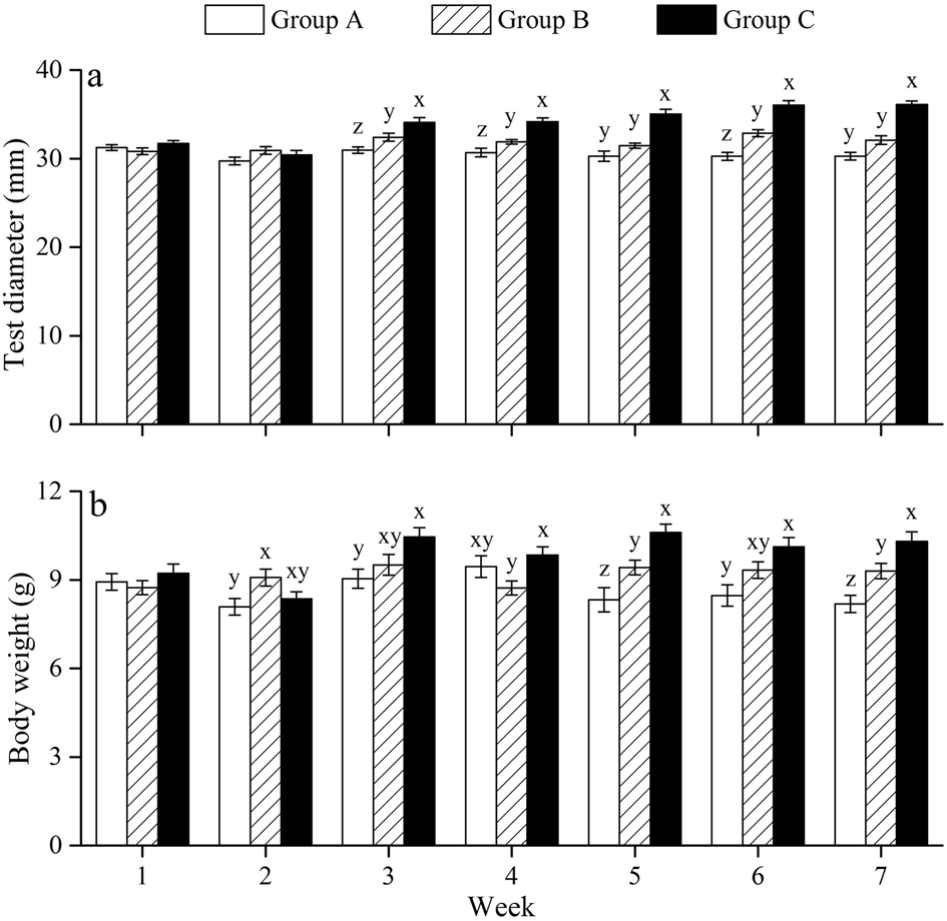
Test diameter (**a**) and wet body weight (**b**) of *Strongylocentrotus intermedius* of the experimental groups in 7 weeks (mean ± SD) (N = 8). Letters above the bars represent significance in each week (*P* < 0.05).

Significant differences in test diameter were found between groups A and B in weeks 3, 4, 6 (all *P* < 0.05). Test diameter in group C was significantly larger than that in group B in weeks 3, 4, 5, 6, 7 (all *P* < 0.05) (Fig. 3a; Supplementary Table 1).

Further, body weight in group B was significantly greater than that in group A in weeks 2, 5, 7 (all *P* < 0.05). Compared with group B, significantly greater body weight occurred in group C in weeks 4, 5, 7 (all *P* < 0.05) (Fig. 3b; Supplementary Table 1).

### Lantern length, lantern weight and gut weight

**Figure 4:**
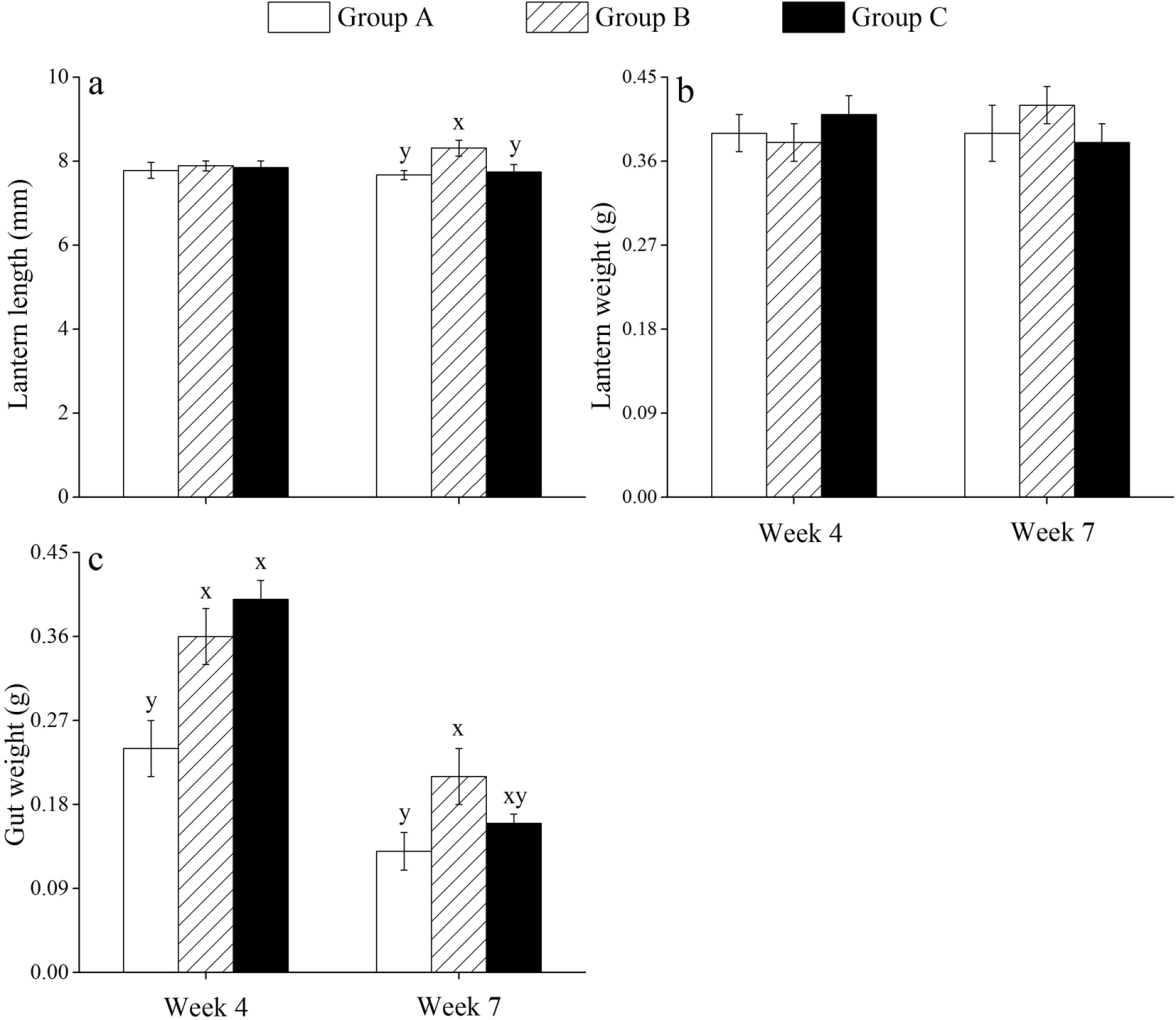
Lantern length (**a**), wet lantern weight (**b**) and wet gut weight (**c**) of *Strongylocentrotus intermedius* of the experimental groups in week 4 and week 7 (mean ± SD, N = 8). Letters above the bars represent significance in each week (*P* < 0.05).

No significant differences were found among groups in lantern length and lantern weight in week 4 (all *P* > 0.05 for lantern length, Fig. 4a) (all *P* > 0.05 for lantern weight, Fig. 4b) (Supplementary Table 2). However, the lantern length in week 7 was significantly greater in group B than that in group A (*P* < 0.05) and in group C (*P* < 0.05) (Fig. 4a; Supplementary Table 2).

There were significant differences in gut weight between groups A and B in week 4 and week 7 (both *P* < 0.05), but not between groups B and C (both *P* > 0.05) (Fig. 4c; Supplementary Table 2).

### Aristotle's lantern reflex

**Figure 5:**
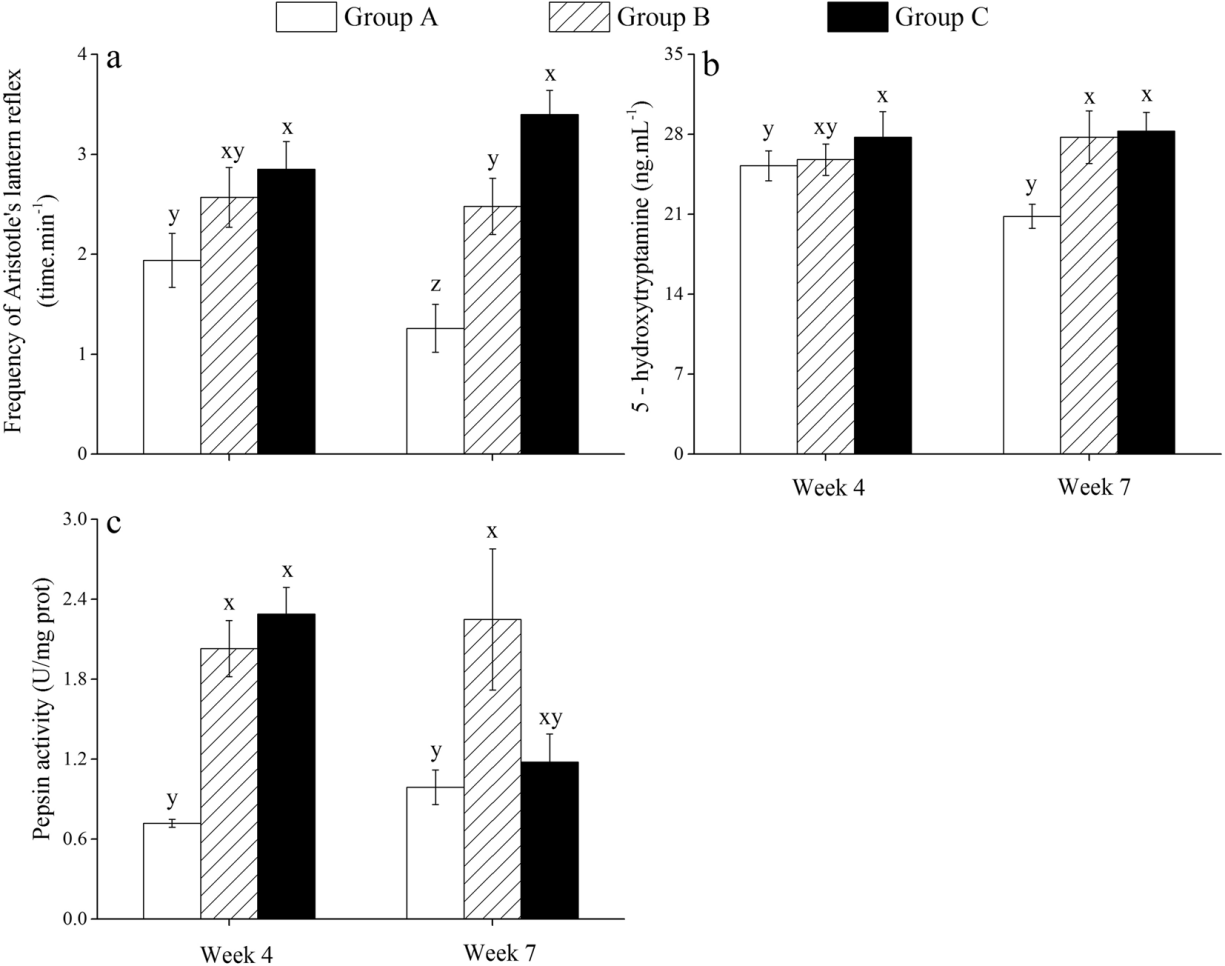
Aristotle’s lantern reflex (**a**), the concentration of 5-hydroxytryptamine (**b**) and pepsin activity (**c**) of *Strongylocentrotus intermedius* of the experimental groups in week 4 and week 7 (mean ± SD, N = 8). Letters above the bars represent significance in each week (*P* < 0.05).

No significant differences in Aristotle's lantern reflex were found neither between groups A nor B, nor groups B and C (both *P* > 0.05) in week 4. Compared with group B, Aristotle's lantern reflex was significantly greater in group C (*P* < 0.05) and lower in group A (*P* < 0.05) in week 7 (Fig. 5a; Supplementary Table 2).

### The concentration of 5-hydroxytryptamine

There was no significant difference in 5-hydroxytryptamine (5-HT) concentration in week 4 between groups A and B (*P* > 0.05), and groups B and C (*P* > 0.05) (Fig. 5b). However, significantly greater 5-HT concentration was found in group B than that in group A (*P* < 0.05) in week 7 (Fig. 5b; Supplementary Table 2).

### Pepsin activity

The pepsin activity of group B was significantly greater than that of group A in week 4 (*P* < 0.05) and week 7 (*P* < 0.05) (Fig. 5c; Supplementary Table 2).

### Gut morphological examination

**Figure 6:**
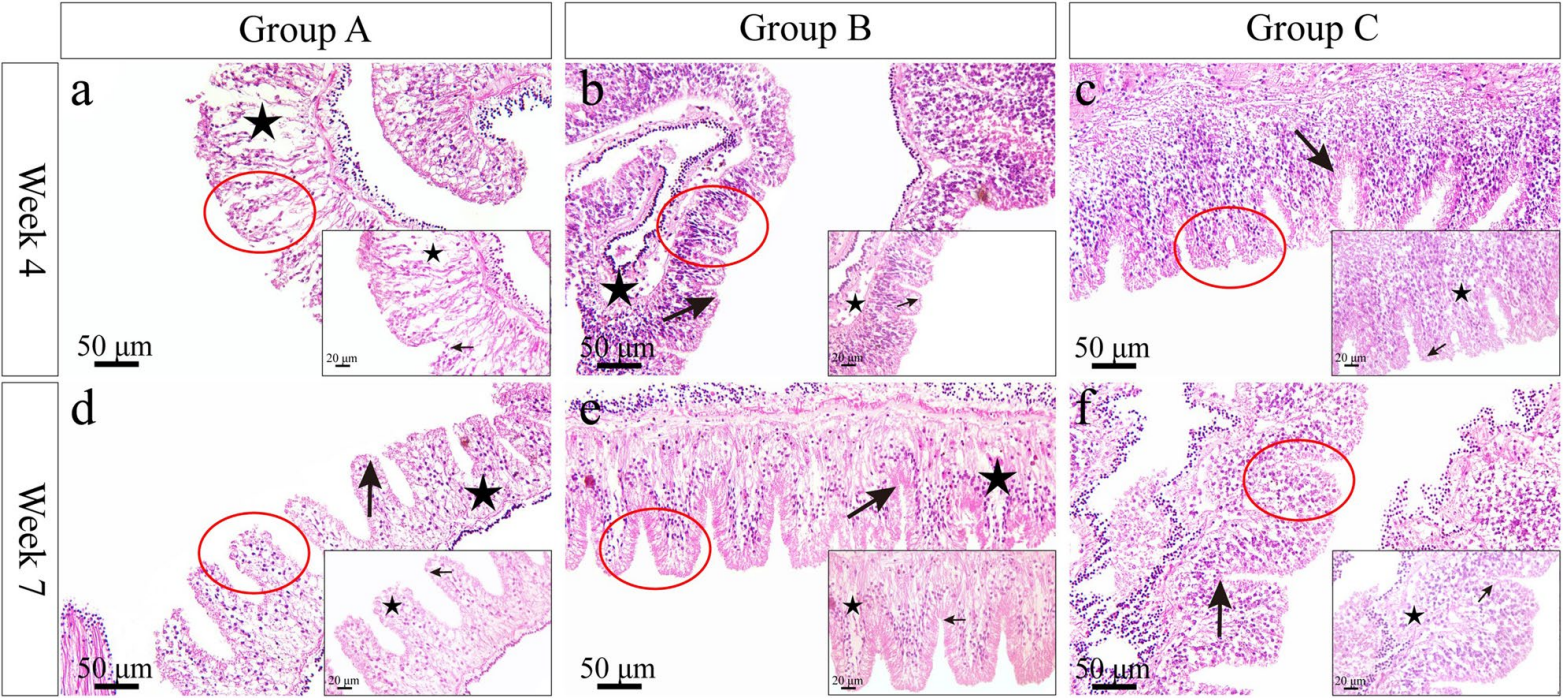
Gut morphology in *Strongylocentrotus intermedius* of the experimental groups in week 4 and week 7. The red circles represent areas for further amplification. Arrows indicate plicae circulars. Stars indicate the hollowing inner structure.

Histological images of gut showed that the plica circulars hollowed greatly and inner microstructures were looser in group A in week 4 (Fig. 6a). Although the tissue cavity improved in week 7, atrophy and deformation of plica circulars were found (Fig. 6d). There were less tissue cavitation and atrophy of plica circulars in the gut of group B in week 4 (Fig. 6b), and cell size greatly increased in week 7 (Fig. 6e). In group C, the plica circulars were arranged tightly and the histology had virtually no tissue cavitation in gut (Fig. 6c, f).

## Discussion

### Multi-layer culture improves food utilization and growth of sea urchins

Methods that maximize somatic growth are crucial to reduce time to market and consequently to improve economic efficiency^25–27^. High water temperature has adverse effects on sea urchin growth^2,28,29^, while little information is available on how to improve sea urchins growth in summer. The present study found that multi-layer cultured sea urchins exhibited significantly larger body size than that without multi-layer culture (the control group), providing experimental evidence for the feasibility of multi-layer culture on sea urchins. Consistently, multi-layer culture received great success in juvenile abalones *Haliotis discus hannai*, where the culture biomass is 6–9 times higher than traditional methods^30^. The consistence is probably due to the similar mobility, feed and energy metabolism between sea urchins and abalones^31,32^. Food utilization is strongly related to the growth in sea urchins^28,33,34^. We observed that multi-layer cultured sea urchins showed significantly better lantern length than that in the control group in week 7. Aristotle's lantern, as a functional flexibility tissue, is important for the food acquisition of sea urchins^35^. They probably have greater ingestion efficiency because of a relatively larger lantern^36^. Significantly superior Aristotle's lantern reflex was consistently found in multi-layer cultured sea urchins than that in the control group in week 7. Aristotle's lantern reflex is the most important behavior conducted by lantern, widely used as an indicator for the capacity of food intake^37,38^. This further indicates that multi-layer culture improves the feeding capacity in sea urchins.

It is important to assess the morphology and pepsin activity of the gut because food digestion is essential for the food utilization of sea urchins. Histological images showed that the plica circulars hollowed seriously and loosed inner microstructures in the control group, while less tissue cavitation occurred in multi-layer cultured sea urchins. Consistently, significantly greater pepsin activity was observed in multi-layer cultured sea urchins than that in the control group. These results indicate that the morphology and structure of the gut are related to the food digestion and absorption efficiency in sea urchins. Pepsin activity is sensitive to culture conditions in aquatic species, including juvenile pomfrets *Pampus argenteus*^39^ and sea urchins^40^. Protein-rich algae, such as *S*. *japonica*^41^ and *Undaria pinnatifido*^42^, are the common diets for *S. intermedius* aquaculture^1^. Therefore, multi-layer culture significantly avoids the negative variation of intestinal histology and improves the pepsin activity, thus probably leads to better digestion of these foods in sea urchins.

The present study further found that eliminating interactions resulted in significantly greater body size and Aristotle's lantern reflex than those were not separated in the multi-layer culture in week 7. Similarly, isolation results in increasing movement and feeding-related behaviors in many species, such as *Drosophila*^43^, adult rats^44^ and desert locust *Schistocerca gregaria*^45^. It suggests that eliminating interactions probably further improve feeding behavior and consequently results in better growth in sea urchins in long-term aquaculture, compared with those not separated in the multi-layer culture. Notably, the present study found that eliminating interactions did not show significantly higher food consumption, pepsin activity and gut weight in the multi-layer culture. This is probably because of the increasing rearing space of multi-layer cultured sea urchins after mass mortality occurred, which probably improved these indicators and subsequently masked the effect of rearing assemblage. Interestingly, the variation tendencies between 5-HT concentration and Aristotle's lantern reflex were largely consistent among sea urchins that were eliminated interactions in the multi-layer culture (group C) and those in the control group (group A). This is consistent with Anstey et al.^46^, who found that long-term solitarious locusts have higher 5-HT concentrations than gregarious individuals. 5-HT, as a neurotransmitter^47^, plays a key role in feeding behavior in organisms, such as the pond snail *Lymnaea stagnalis*^48–50^. It has been well documented that 5-HT is involved in changing behaviors (e.g. aggression, status and courtship) after interactions in many species^51–53^. Collectively, it is likely that sea urchins being less disturbed (i.e. eliminating interactions in the multi-layer culture) during the experiment suffered less stress and consequently produced more 5-HT, improved feeding efficiency and growth.

### Multi-layer culture conditions reduced the morbidity of sea urchins

Mass mortality and morbidity occurred among groups after the sampling in week 4, which was consistent with the high mortality (over 95%) observed in field cultured *S*. *intermedius* in Dalian (121° 45′ N, 38° 82′ E) during our experimental period. This suggests that frequent management probably has a negative impact on the survival of sea urchins in longline culture in summer. The present study found that multi-layer cultured sea urchins (group B) displayed significantly less morbidity than that in the control group (group A) after week 4, suggesting that multi-layer culture can reduce morbidity at medium to long-term. Further, eliminating interactions in multi-layer culture (group C) significantly reduced morbidity compared to the control group (group A) in each week, suggesting that eliminating interactions in multi-layer culture is an effective approach to decreasing disease spread. Spotting disease outbreaks in *S*. *intermedius* at high temperatures (> 20 °C)^54^ and the pathogens are mainly opportunistic bacteria as the genus *Vibrio*, *Aeromonas* and *Flexibacter*^54,55^. Dead animals probably increase the reproduction of pathogens because they are ephemeral resources that provide nutrients for pathogenic bacteria^56,57^. This is consistent with the finding of Stroeymeyt et al.^22^ that the ant *Lasius niger* showed segregation between potential disease sources (e.g. foragers) and high-value individuals (e.g. the queen) to decreases disease transmission. There was no significant difference in morbidity between group C and group B after week 4. A reasonable explanation is that the increasing rearing space in group B probably masked the effect of rearing assemblage bewteen groups B and C. For example, almost three times more the rearing space was found in multi-layer culture (608.8 ± 217.8 cm^3^ ind^−1^) than that in the control group (262.2 ± 137.5 cm^3^ ind^−1^) in week 7. Thus, the segregation (group C) and/or increased rearing space after mass mortality (group B) probably avoid/reduce the physical contacts among the healthy and diseased/dead sea urchins and consequently decrease disease spread. Unfortunately, significant reductions in mortality were not observed in both multi-layer culture conditions. Exploring an effective approach to avoiding mass mortality in summer is thus urgent for the longline culture of sea urchins.

## Materials and methods

### Sea urchins and experimental design

Seven hundred small *S*. *intermedius* (31.9 ± 0.4 mm of test diameter, mean ± SD) were chosen from an aquaculture farm in Changhai County, Dalian (122° 63′ N, 39° 25′ E) on 23 July 2020. They were subsequently transported to the Key Laboratory of Mariculture and Stock Enhancement in North China's Sea, Ministry of Agriculture and Rural Affairs at Dalian Ocean University (121° 56′ N, 38° 87′ E) and maintained in a fiberglass tank (a closed culture system, length × width × height: 150 × 100 × 60 cm) with aeration for 7 days to acclimatize to laboratory conditions. The kelp *Saccharina japonica*, which is the most common food used for *S*. *intermedius* culture^58^, was fed ad libitum under the neutral photoperiod (12 h light:12 h dark). One-half of the seawater was changed daily. Water temperature, pH and salinity were 22.6 ± 0.2 °C, 7.7 ± 0.3 and 30.7 ± 0.1 ‰ (Mean ± SD) according to the daily measurement using a portable water quality monitor (YSI Incorporated, OH, USA), respectively.

**Figure 7:**
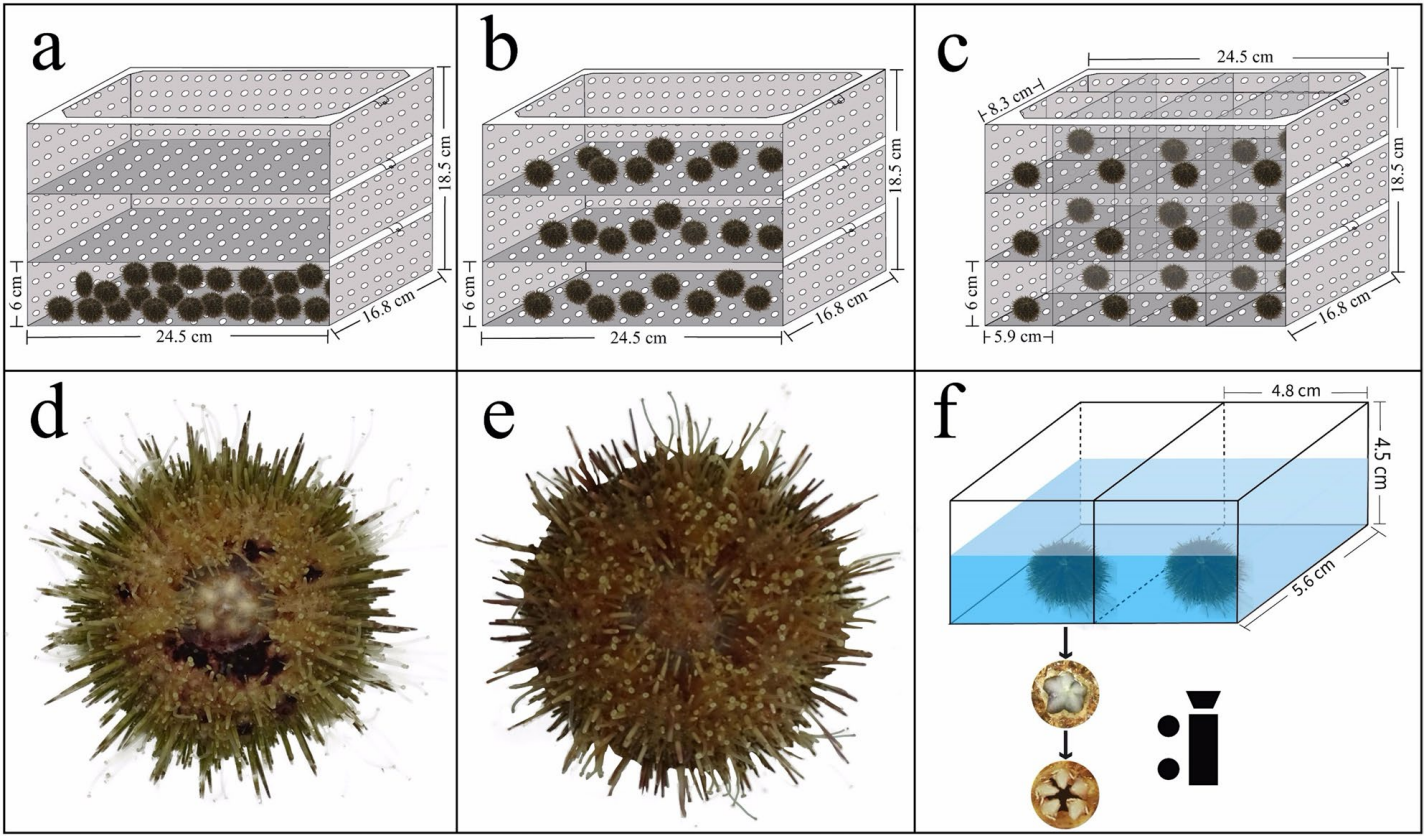
Diagrams of the experimental cages used for the groups A (**a**), B (**b**) and C (**c**), the sea urchin with the spotting disease (**d**) and without the disease (**e**) and the devices used for measuring the Aristotle's lantern reflex (**f**).

The rearing space was defined as the ratio of culture volume to the number of sea urchins (cm^3^ ind^−1^). Rearing assemblage is the main factor being tested in this study. To simulate the currently used rearing assemblage in longline culture, 24 individuals were placed at plastic devices without layer divisions (length × width × height: 24.5 × 16.8 × 6 cm for culture volume; 25 holes of 0.5 cm diameter/100 cm^2^) as group A (the control group, 102.9 cm^3^ ind^−1^ of initial rearing space, Fig. 7a). To investigate whether multi-layer rearing assemblage improves the survival, food utilization and growth, 24 sea urchins were equally put into the cages where were evenly divided into three layers (8 sea urchins in each layer and length × width × height: 24.5 × 16.8 × 6 cm for each layer, 308.7 cm^3^ ind^−1^ of initial rearing space; 25 holes of 0.5 cm diameter/100 cm^2^; group B; Fig. 7b). Further, to evaluate whether eliminating interaction further contributes to the improvement of these commercially important traits of sea urchins in multi-layer rearing assemblage, 8 sea urchins were divided into eight divisions for each layer in the cages as group C (length × width × height: 8.3 × 5.9 × 6 cm for each division, 297.36 cm^3^ ind^−1^ of initial rearing space; 25 holes of 0.5 cm diameter/100 cm^2^; Fig. 7c). Each treatment had 8 replicates. All devices were placed in a fiberglass tank (length × width × height: 150 × 100 × 60 cm) and immersed in water for ~ 30 cm with aeration. They were easily disassembled for the experimental management.

The experimental period was about ~ 7 weeks (from 31 July 2020 to 20 September 2020) under the neutral photoperiod (12 h light: 12 h dark). The kelp, which was regularly collected in the intertidal waters at Heishijiao, Dalian (121° 58′ E, 38° 87′ N), was daily provided to sea urchins in abundance for all the groups. The remained kelp, feces and dead sea urchins were removed daily. One-half of the seawater was replaced daily by the fresh and filtered seawater which was pumped from the coast of Heishijiao, Dalian. Water temperature was not controlled, ranging from 22.2 to 24.5 °C (the natural seasonal cycle of increasing temperature during summer in the region). Water quality parameters were measured weekly as salinity 29.3 ± 0.6 ‰, pH 7.8 ± 0.2 (mean ± SD) using a portable water quality monitor (YSI Incorporated, OH, USA).

To ensure the random sampling, sea urchins were taken out from the experimental device and placed in 24 plastic boxes (labeled from number 1 to number 24, length × width × height: 6 × 6 × 4 cm for each box). Individuals were chosen corresponding to the number (within 24) generated by the “sample” function in R studio (1.1.463). Sampling was re-conducted if the number corresponds to empty, dead or diseased sea urchins.

### Mortality and morbidity

Spotting disease, which appears as spotting lesions with red, purple or blackish color on the test (Fig. 7d), is the most common lethal disease in *S*. *intermedius* aquaculture^12^. Sea urchin without disease is shown in Fig. 7e. Dead sea urchins were removed daily and the number of survivor and diseased sea urchins was recorded weekly for each cage during the experiment (N = 8).

### Food consumption

The measurement of food consumption (g dry weight) was conducted once a week (24 h from Tuesday to Wednesday) (N = 8). The total supplied and remained diets were weighted wet by an electric balance (G & G Co., San Diego, USA) after the removal of the surface moisture. The dried weights of feces and samples of supplied and uneaten kelp were determined after 4 days at 80 °C in a convection oven (Yiheng Co., Shanghai, China).

Food consumption was calculated as follows (revised from Hu et al.^9^ for being more concise):

$${\text{F}} = \frac{{{\text{A}}_{0} \times \frac{{{\text{A}}_{1} }}{{{\text{A}}_{2} }} - {\text{B}}_{0} \times \frac{{{\text{B}}_{1} }}{{{\text{B}}_{2} }}}}{{\text{N}}}$$

F = dry food intake per sea urchin (g ind^−1^ day^−1^), A_0_ = wet weight of total supplied diets (g), B_0_ = wet weight of total uneaten diets (g), A_1_ = dried weight of sample supplied diets (g), A_2_ = wet weight of sample supplied diets (g), B_1_ = dry weight of sample uneaten diets (g), B_2_ = wet weight of sample uneaten diets (g), N = the number of sea urchins.

### Growth

Test diameter and lantern length were measured using a digital vernier caliper (Mahr Co., Ruhr, Germany). Body, lantern and gut were weighted wet using an electric balance (G & G Co., San Diego, USA). Test diameter and body weight were evaluated every Wednesday. The average value of the three individuals was considered as the trait value for each replicate (N = 8). Lantern length, wet lantern weight and wet gut weight were recorded in week 4 (29 August 2020) and week 7 (20 September 2020) (N = 8).

### Aristotle's lantern reflex

Aristotle's lantern reflex, which refers to one cycle from the opening to the closing of the teeth^59^, was measured using a simple device according to the method of Ding et al.^38^. There were small compartments (length × width × height: 4.8 × 5.6 × 4.5 cm) with a film (made by 3 g agar and 2 g kelp powder) on the bottom of the device^38^ (Fig. 7f). The frequency of Aristotle's lantern reflex was counted within 5 min using a digital camera (Canon Co., Shenzhen, China) under the device in week 4 (29 August 2020) and week 7 (20 September 2020). The average value of all the 5 individuals was considered as Aristotle's lantern reflex for each replicate (N = 8).

### 5-HT concentration

The 5-HT is a signaling molecule, playing an important role in regulating feeding behavior^52^. To evaluate whether 5-HT is involved in Aristotle's lantern reflex, 5-HT concentration of muscle in lantern was measured for each treatment in week 4 and week 7. 5-HT concentration was considered as the average value of all the 3 healthy individuals for each replicate (N = 8).

The concentration of 5-HT was measured using ELISA kits (Nanjing Jiancheng Bio-engineering Institute, Nanjing, China) according to the instructions of the manufacturer. After adding the enzyme-labeled antibody, the substrate became a colored product that was directly related to the amount of the substance tested. The concentrations of 5-HT were calculated by comparing the optical density (O.D.) value of the samples to the standard curve and calculated according to the following formula (according to the kit's instructions):

$${\text{Y}} = \frac{1}{{({\text{a }} + {\text{bx}}^{{\text{c}}} )}}$$

Y = the concentration of 5-HT (ng mL^−1^), x = the O.D. value of the samples, a = 0.00027, b = 0.12086, c = 1.36806.

### Pepsin activity

Pepsin is important for sea urchins to digest protein-rich algae^40,60^. Pepsin activity was analyzed using the pepsin kits (Nanjing Jiancheng Bio-engineering Institute, Nanjing, China) in week 4 and week 7, following the instructions of the manufacturer. The average value of all the 3 individuals was considered as the pepsin activity for each replicate (N = 8). The procedures include enzyme reaction and color development reaction^39^. The temperature of reaction was 37 °C and pepsin activities were counted as U mg protein^−1^. The formula of pepsin activity is shown as follows (according to the kit's instructions):

$${\text{P}} = \frac{{{\text{M}}_{0} - {\text{M}}_{1} }}{{{\text{M}}_{2} - {\text{M}}_{3} }} \times \frac{{{\text{S}}_{0} }}{{{\text{S}}_{1} }} \times \frac{{{\text{V}}_{1} \times {\text{V}}_{2} }}{{{\text{V}}_{3} }}$$

P = pepsin activity (U/mg prot), M_0_ = the O.D. value of the sample, M_1_ = the O.D. value of comparison, M_2_ = the standard O.D. value, M_3_ = blank O.D. value, S_0_ = the standard concentration (50 μg mL^−1^), S_1_ = reaction time (10 min), V_1_ = total volume of reaction solution (0.64 mL), V_2_ = sample protein concentration (0.04 mL), V_3_ = sampling volume (mg prot/mL).

### Gut morphological examination

After sea urchins were dissected on week 4 and week 7, all gut tissue samples (~ 1 g for each sample) were fixed in Bouin's solution (glacial acetic acid: formaldehyde: saturated picric acid solution = 1:5:15) according to the method of Wu et al.^61^. They were subsequently transferred for gradient dehydration, embedding, cutting, staining and observation^62^ (N = 24).

### Statistical analysis

Kolmogorov–Smirnov test and Levene test were used to analyze the normal distribution and homogeneity of the data, respectively. Rearing assemblage was set as the main factor in the one-way ANOVA with three levels: the control system without layer divisions (group A), a second system with divisions in the cages to simulate the three layers cages (group B) and a third system with individual divisions for each sea urchin (group C). One-way ANOVA was used to analyze the mortality (in weeks 3, 4, 5, 6, 7), morbidity (in weeks 3, 6, 7), food consumption (in weeks 2, 5, 7), test diameter (in weeks 1, 2, 3, 4, 5, 6), body weight (in weeks 1, 4, 5, 7), 5-HT, pepsin activity, lantern length, lantern weight and gut weight. Duncan multiple comparison analysis was performed when significant differences were found in the one-way ANOVA. Kruskal–Wallis test was carried out to compare the differences of mortality (weeks 1, 2), morbidity (weeks 1, 2, 4, 5), food consumption (weeks 1, 3, 4, 6), test diameter (week 7), body weight (weeks 2, 3, 6) and Aristotle's lantern reflex, because of non-normal distribution and/or heterogeneity of variance. A non-parametric post-hoc test was carried out when significant differences were found in the Kruskal–Wallis test. All data analyses were performed using SPSS 19.0 statistical software. A probability level of *P* < 0.05 was considered significant.

## Supplementary Information

Supplementary Tables.
