# Supplementary material for: Effects of eliminating interactions in multi-layer culture on survival, food utilization and growth of small sea urchins Strongylocentrotus intermedius at high temperatures
Source: Sci Rep. 2021 Jul 23;11:15116. doi: 10.1038/s41598-021-94546-1 (PMC8302603; doi:10.1038/s41598-021-94546-1)
Supplement: Supplementary file 1 — Supplementary Tables. [file 41598_2021_94546_MOESM1_ESM.docx]

**Supporting information**

**Article title:** Effects of eliminating interactions in multi-layer culture on survival, food utilization and growth of small sea urchins *Strongylocentrotus intermedius* at high temperatures

**Author list:** Fangyuan Hu, Xiaomei Chi, Mingfang Yang, Peng Ding, Donghong Yin, Jingyun Ding, Xiyuan Huang, Jia Luo, Yaqing Chang^*^, Chong Zhao^*^

**^*^Email:** changlab@hotmail.com (Chang Y), chongzhao@dlou.edu.cn (Zhao C)

**Supplementary Table 1.** Mortality, morbidity, food consumption, test diameter and body weight of sea urchins and their statistical comparisons in the experiment.

|  |  | Week 1 | | | Week 2 | | | Week 3 | | | Week 4 | | | Week 5 | | | Week 6 | | | Week 7 | | |
| --- | --- | --- | --- | --- | --- | --- | --- | --- | --- | --- | --- | --- | --- | --- | --- | --- | --- | --- | --- | --- | --- | --- |
|  |  | N | Kruskal–Wallis *H* | *P* | N | Kruskal–Wallis *H* | *P* | N | *F* | *P* | N | *F* | *P* | N | *F* | *P* | N | *F* | *P* | N | *F* | *P* |
| Mortality | A-B | 8 | 11.284 | 0.014 | 8 | 12.588 | 0.017 | 8 | 4.182 | 1.000 | 8 | 11.448 | 0.579 | 8 | 0.406 | 0.591 | 8 | 0.406 | 0.591 | 8 | 0.148 | 0.661 |
|  | A-C |  |  | 1.000 |  |  | 1.000 |  |  | 0.021 |  |  | <0.001 |  |  | 0.382 |  |  | 0.382 |  |  | 0.626 |
|  | B-C |  |  | 0.009 |  |  | 0.003 |  |  | 0.021 |  |  | 0.001 |  |  | 0.732 |  |  | 0.732 |  |  | 0.961 |
|  |  | N | Kruskal–Wallis *H* | *P* | N | Kruskal–Wallis *H* | *P* | N | *F* | *P* | N | Kruskal–Wallis *H* | *P* | N | Kruskal–Wallis *H* | *P* | N | *F* | *P* | N | *F* | *P* |
| Morbidity | A-B | 8 | 12.729 | 1.000 | 8 | 14.861 | 0.866 | 8 | 19.167 | 0.071 | 8 | 17.768 | 0.305 | 8 | 11.225 | 0.015 | 8 | 11.694 | 0.001 | 8 | 17.616 | <0.001 |
|  | A-C |  |  | 0.009 |  |  | 0.001 |  |  | <0.001 |  |  | <0.001 |  |  | 0.008 |  |  | <0.001 |  |  | <0.001 |
|  | B-C |  |  | 0.004 |  |  | 0.022 |  |  | <0.001 |  |  | 0.033 |  |  | 1.000 |  |  | 0.754 |  |  | 0.580 |
|  |  | N | Kruskal–Wallis *H* | *P* | N | *F* | *P* | N | Kruskal–Wallis *H* | *P* | N | Kruskal–Wallis *H* | *P* | N | *F* | *P* | N | Kruskal–Wallis *H* | *P* | N | *F* | *P* |
| Food consumption | A-B | 8 | 5.526 | 0.063 | 8 | 39.19 | 0.175 | 8 | 2.288 | 0.319 | 8 | 7.087 | 0.915 | 8 | 14.48 | 0.573 | 8 | 6.145 | 0.040 | 8 | 0.502 | 0.538 |
|  | A-C |  |  |  |  |  | <0.001 |  |  |  |  |  | 0.024 |  |  | <0.001 |  |  | 0.487 |  |  | 0.719 |
|  | B-C |  |  |  |  |  | <0.001 |  |  |  |  |  | 0.018 |  |  | <0.001 |  |  | 0.847 |  |  | 0.333 |
|  |  | N | *F* | *P* | N | *F* | *P* | N | *F* | *P* | N | *F* | *P* | N | *F* | *P* | N | *F* | *P* | N | Kruskal–Wallis *H* | *P* |
| Test diameter | A-B | 8 | 1.766 | 0.372 | 8 | 1.703 | 0.070 | 8 | 12.007 | 0.027 | 8 | 18.662 | 0.040 | 8 | 27.379 | 0.082 | 8 | 39.986 | <0.001 | 8 | 43.782 | 0.088 |
|  | A-C |  |  | 0.331 |  |  | 0.302 |  |  | <0.001 |  |  | <0.001 |  |  | <0.001 |  |  | <0.001 |  |  | <0.001 |
|  | B-C |  |  | 0.065 |  |  | 0.462 |  |  | 0.010 |  |  | <0.001 |  |  | <0.001 |  |  | <0.001 |  |  | <0.001 |
|  |  | N | *F* | *P* | N | Kruskal–Wallis *H* | *P* | N | Kruskal–Wallis *H* | *P* | N | *F* | *P* | N | *F* | *P* | N | Kruskal–Wallis *H* | P | N | *F* | *P* |
| Body weight | A-B | 8 | 0.722 | 0.632 | 8 | 7.083 | 0.026 | 8 | 10.501 | 1.000 | 8 | 3.428 | 0.098 | 8 | 12.555 | 0.020 | 8 | 11.580 | 0.142 | 8 | 13.409 | 0.008 |
|  | A-C |  |  | 0.478 |  |  | 0.998 |  |  | 0.006 |  |  | 0.368 |  |  | <0.001 |  |  | 0.002 |  |  | <0.001 |
|  | B-C |  |  | 0.237 |  |  | 0.289 |  |  | 0.055 |  |  | 0.012 |  |  | 0.011 |  |  | 0.481 |  |  | 0.016 |

A, B and C mean the control group, multi-layer culture and segregation in multi-layer culture, respectively. A-B, A-C and B-C mean the comparisons between groups A and B, groups A and C, groups B and C, respectively.

**Supplementary Table 2.** Lantern length, lantern weight, gut weight, Aristotle's lantern reflex, 5-HT concentration and pepsin activity of sea urchins and their statistical comparisons in week 4 and week 7.

|  |  | Week 4 | | | Week 7 | | |
| --- | --- | --- | --- | --- | --- | --- | --- |
|  |  | N | *F* | *P* | N | *F* | *P* |
| Lantern length | A-B | 8 | 0.141 | 0.606 | 8 | 4.61 | 0.011 |
|  | A-C |  |  | 0.739 |  |  | 0.754 |
|  | B-C |  |  | 0.855 |  |  | 0.023 |
|  |  | N | *F* | *P* | N | *F* | *P* |
| Lantern weight | A-B | 8 | 0.532 | 0.698 | 8 | 0.545 | 0.430 |
|  | A-C |  |  | 0.536 |  |  | 0.863 |
|  | B-C |  |  | 0.318 |  |  | 0.339 |
|  |  | N | *F* | *P* | N | *F* | *P* |
| Gut weight | A-B | 8 | 10.947 | 0.002 | 8 | 3.159 | 0.020 |
|  | A-C |  |  | <0.001 |  |  | 0.262 |
|  | B-C |  |  | 0.333 |  |  | 0.189 |
|  |  | N | Kruskal–Wallis *H* | *P* | N | Kruskal–Wallis *H* | *P* |
| Aristotle's lantern reflex | A-B | 8 | 6.649 | 0.373 | 8 | 24.114 | 0.017 |
|  | A-C |  |  | 0.033 |  |  | <0.001 |
|  | B-C |  |  | 0.784 |  |  | 0.049 |
|  |  | N | *F* | *P* | N | *F* | *P* |
| 5-HT concentration | A-B | 8 | 2.712 | 0.154 | 8 | 5.651 | 0.011 |
|  | A-C |  |  | 0.032 |  |  | 0.007 |
|  | B-C |  |  | 0.421 |  |  | 0.829 |
|  |  | N | *F* | *P* | N | *F* | *P* |
| Pepsin activity | A-B | 8 | 11.684 | 0.001 | 8 | 3.658 | 0.034 |
|  | A-C |  |  | 0.001 |  |  | 0.727 |
|  | B-C |  |  | 0.362 |  |  | 0.050 |

A, B and C mean the control group, multi-layer culture and segregation in multi-layer culture, respectively. A-B, A-C and B-C mean the comparisons between groups A and B, groups A and C, groups B and C, respectively.
